# The effectiveness of nurse led health guidance in improving self-management skills, disease awareness, and quality of life for outpatient patients

**DOI:** 10.3389/fmed.2026.1774746

**Published:** 2026-07-16

**Authors:** Wenjing Wang, Yan Wang, Ying Xu

**Affiliations:** 1 Outpatient Department of Yongkang First People’s Hospital, Yongkang, China; 2 Nursing Department, Yongkang First People’s Hospital, Yongkang, China

**Keywords:** disease awareness, effectiveness, nurse led health guidance, outpatient patients, quality of life, self-management skills

## Abstract

**Background:**

The growing burden of chronic diseases like asthma necessitates effective self-management strategies to improve patient outcomes. Despite advancements in asthma treatment, many patients struggle with disease awareness and self-management, leading to impaired quality of life. Nurse-led health guidance has shown potential in enhancing patient education and management across chronic conditions, but its specific impact on outpatient asthma management remains under-researched.

**Methods:**

This retrospective cohort study evaluated 159 stable asthma outpatients from January 2021 to January 2023 at Yongkang First People’s Hospital. Participants were divided into a control group (*n* = 71) receiving standard care with traditional education and an intervention group (*n* = 88) receiving standard care supplemented by a 12-week structured nurse-led health guidance programme delivered through weekly face-to-face sessions and biweekly telephone follow-ups. Baseline characteristics were compared, and outcomes measured included health literacy, self-care abilities, disease awareness, quality of life, and patient satisfaction. Between-group differences in post-intervention scores were examined using analysis of covariance (ANCOVA) adjusting for baseline values, and multivariable linear regression was performed to account for potential confounders. Effect sizes (Cohen’s *d*) with 95% confidence intervals were reported.

**Results:**

Baseline characteristics were similar between groups. Post-intervention, the intervention group showed significant improvements in health literacy (NVS, TOFHLA, HLS-EU, eHEALS scores), self-care abilities, and disease awareness (AKQ scores) compared to controls after adjusting for baseline values and potential confounders (*p* < 0.05). Quality of life indices (SF-36 scores) and patient satisfaction were also significantly higher in the intervention group (*p* < 0.05). These associations persisted after multivariable adjustment for age, sex, education level, disease duration, and asthma severity.

**Conclusion:**

Nurse-led health guidance is associated with substantially improved self-management skills, disease awareness, and quality of life in asthma outpatients, underscoring its potential value in chronic disease management. Future prospective randomized studies are warranted to confirm these findings.

## 1 Introduction

The growing prevalence of chronic diseases, including asthma, poses a significant challenge to health care systems worldwide (1). Asthma, a chronic respiratory condition characterized by airway inflammation and hyper-responsiveness, affects millions globally, necessitating ongoing medical management and self-care strategies (2, 3). Despite advancements in pharmacotherapy, optimal asthma management remains elusive for many patients, often due to inadequate self-management skills and insufficient disease awareness (4). The inability of patients to effectively manage their conditions is associated with frequent exacerbations, impaired quality of life, and increased health system burdens (5, 6). In response to these challenges, health care professionals are increasingly recognizing the importance of patient education and self-management support as integral components of chronic disease management (7). Notably, smoking cessation remains a cornerstone of asthma management, as continued tobacco exposure is associated with accelerated lung function decline, increased exacerbation frequency, and reduced treatment responsiveness (8).

Nurses, as the largest segment of health care professionals, are uniquely positioned to provide continuous and structured health guidance (9). Their role extends beyond basic care provision to encompass patient education, empowerment, and support, which are vital for effective chronic disease management (10). Nurse-led health interventions have shown promise in improving self-management skills and health outcomes across various chronic conditions (11, 12). A Cochrane systematic review on self-management education for adults with asthma demonstrated that structured educational programmes, including those delivered by nurses, can lead to improved health outcomes, reduced emergency visits, and better quality of life (13). However, the specific impact of nurse-led health guidance on asthma patients, particularly in outpatient settings, warrants deeper investigation (14). Understanding the potential of such interventions could enhance asthma management strategies, bridging existing gaps in patient care (15).

Despite the potential of nurse-led interventions, research in this domain remains limited, particularly studies exploring their impact in outpatient settings (16). Most existing evidence focuses on acute care or inpatient interventions, with scarce attention on outpatient management (17). Bridging this research gap could lead to improved care models that integrate nurse-led strategies in routine asthma management (18). The implications are broad, offering insights that could be applied to various chronic diseases characterized by similar management challenges (19).

In this retrospective cohort study, we aimed to evaluate the association between nurse-led health guidance and self-management skills, disease awareness, and quality of life among outpatients with asthma. By examining the relationship between structured, nurse-led interventions and these domains, this study seeks to provide observational evidence supporting the integration of nurse-led health guidance into chronic disease management strategies. The findings may inform health policy considerations and inspire further prospective research into nurse-led interventions tailored for outpatient settings, ultimately contributing to better health outcomes for patients with asthma and beyond.

## 2 Materials and methods

### 2.1 Case selection

This retrospective cohort study included 159 asthma patients in a stable condition, who were treated as outpatients at Yongkang First People’s Hospital from January 2021 to January 2023. Patient demographic information was collected through case files, encompassing general data, lung examinations, health literacy scores, disease cognition scores, self-management scores, quality of life scores, and satisfaction levels. Because this retrospective study utilized de-identified patient data extracted from existing medical records, there was no risk of additional harm or impact on patient care. Therefore, individual written informed consent was waived by the Ethics Review Board. This waiver and the study itself were approved by the institution’s Ethics Review Board and Ethics Committee (approval number: ZJ-YK-012), conforming to the Declaration of Helsinki and regulatory guidelines for retrospective studies.

### 2.2 Inclusion and exclusion criteria

Inclusion criteria were as follows: (1) Patients over the age of 18, capable of understanding and cooperating with various treatments and examinations; (2) Diagnosed with asthma according to the Global Initiative for Asthma (GINA) 2020 guidelines, defined as a history of variable respiratory symptoms such as wheeze, shortness of breath, chest tightness, and cough that vary over time and in intensity, together with variable expiratory airflow limitation confirmed by bronchodilator reversibility testing (increase in FEV1 >12% and >200 mL from baseline after administration of 200–400 μg salbutamol) (20); (3) Stable vital signs with no exacerbation in the preceding 4 weeks; (4) Informed and voluntary participation in the study.

Exclusion criteria included: (1) Patients with other severe progressive diseases; (2) Those with neurological disorders or cognitive impairments; (3) Individuals unable to complete the questionnaire survey for any reason during the study.

### 2.3 Grouping and intervention methods

Grouping criteria: Participants were divided into two groups based on their choice of health guidance model: the control group, comprising 71 individuals, and the intervention group, consisting of 88 individuals. The control group received standard medical treatment in accordance with GINA guidelines, including inhaled corticosteroids with or without long-acting beta-agonists titrated to asthma severity, along with traditional education consisting of routine verbal medication instructions and a standardized asthma information leaflet provided at each outpatient visit. The intervention group received identical standard medical treatment supplemented by a structured 12-week nurse-led health guidance programme. The programme was delivered by two registered nurses with over 5 years of respiratory nursing experience who had completed a 16-h standardized training course covering motivational interviewing techniques, asthma pathophysiology education, and self-management coaching. Each patient in the intervention group received weekly 30-min face-to-face individual counselling sessions at the outpatient clinic and biweekly 15-min structured telephone follow-ups. The programme content encompassed four core modules: disease awareness education (asthma pathophysiology, trigger identification, and recognition of exacerbation warning signs), medication guidance (inhaler technique demonstration with return demonstration, adherence monitoring, and action plan development), psychological care (anxiety and stress management strategies, coping skills training), and lifestyle counselling (dietary advice, physical activity recommendations, and smoking cessation counselling where applicable). Programme fidelity was monitored through a standardized checklist completed by nurses after each session, and a senior respiratory physician conducted monthly audits of a random sample of 10% of session records. As participants self-selected into groups based on their preference for the health guidance model, this study is observational in nature and the findings should be interpreted with appropriate caution regarding causal inference.

### 2.4 Pulmonary function testing

A comprehensive lung function analyzer (Jaeger, Inc., Bodnegg, Germany) was utilized to assess various pulmonary function indicators. The study involved comparing these indicators between the two groups of patients before and after treatment. Key measurements included the forced expiratory volume in one second (FEV1), forced vital capacity (FVC), peak expiratory flow rate (PEFR), and total lung capacity (TLC), among others.

### 2.5 Health literacy evaluation

The Newest Vital Sign (NVS) is a swift screening tool designed to assess functional health literacy (21). The validated Chinese version of the NVS was used in this study. It measures an individual’s health literacy by evaluating their ability to interpret and comprehend nutritional labels. The test involves a straightforward graphic, such as the nutrition label on an ice cream container, and requires participants to answer six questions based on the information presented. These questions may involve calculating, comparing, or interpreting nutritional components. The scoring ranges from 0 to 6, with higher scores indicating greater health literacy. A score of 0-1 suggests high likelihood of limited literacy, 2-3 indicates possible limited literacy, and 4–6 suggests adequate literacy. The Cronbach’s alpha for the NVS in the original validation study is 0.74 (21), and in the present sample it was 0.76, indicating acceptable reliability.

The Test of Functional Health Literacy in Adults (TOFHLA) is a standardized assessment used to evaluate adults’ ability to read and comprehend health-related information (22). The validated Chinese version (S-TOFHLA) was administered. TOFHLA comprises two components: reading comprehension (36 items) and numeracy (17 items), yielding a total score ranging from 0 to 100. Scores of 0–59 indicate inadequate health literacy, 60–74 indicate marginal health literacy, and 75–100 indicate adequate health literacy. The TOFHLA has a Cronbach’s alpha of 0.943 in the original validation study (22), and in the present sample it was 0.91, indicating excellent reliability.

The Health Literacy Survey—European Union (HLS-EU-Q47) is a standardized tool designed to assess an individual’s skills and confidence in managing health information (23). The validated Chinese adaptation (HLS-EU-Q47-CN) was used in the present study. The full questionnaire contains 47 items across three domains: healthcare, disease prevention, and health promotion. Each item is rated on a 4-point scale from “very difficult” to “very easy,” yielding a total score from 0 to 50 after standardized transformation. Higher scores indicate greater health literacy. The HLS-EU-Q has a Cronbach’s alpha of 0.84 in the original validation study (23), and in the present sample it was 0.82, indicating high reliability.

The Electronic Health Literacy Scale (eHEALS) serves as a quantitative measure to assess an individual’s ability to locate, comprehend, and utilize health information on the Internet (24). The validated Chinese version of the eHEALS was used. The questionnaire comprises 8 core items rated on a 5-point Likert scale ranging from “strongly disagree” to “strongly agree,” yielding total scores from 8 to 40. Additionally, two supplementary items assess perceived usefulness and importance of online health resources, bringing the extended score range to 10–50. A higher score indicates greater electronic health literacy. The eHEALS demonstrates a Cronbach’s alpha of 0.94 in the original validation study (24), and in the present sample it was 0.92, indicating excellent reliability.

### 2.6 Self care ability assessment

The Exercise of Self-Care Agency (ESCA) scale, developed by Kearney and Fleischer, was employed to evaluate patients’ self-care abilities (25). The validated Chinese version was used. The ESCA assesses four dimensions: self-concept (12 items), self-care knowledge (17 items), self-preservation awareness (6 items), and self-care skills (8 items), totalling 43 items. Each item uses a 5-point Likert scale (0 = very uncharacteristic to 4 = very characteristic), with 11 items reverse-scored. Higher total scores indicate a greater capacity for self-care. The scale demonstrates a reliability coefficient of 0.87 in the original validation study (25), and in the present sample it was 0.85, indicating good internal consistency.

### 2.7 Disease awareness level

The Asthma Knowledge Questionnaire (AKQ) serves as a tool to evaluate asthma patients’ understanding of disease management (26). The validated Chinese version was administered. Comprising 30 true/false items, the AKQ offers a maximum score of 30, testing patients on their knowledge of asthma pathophysiology, treatment strategies, trigger awareness, and symptom control. A score above 24 reflects a strong grasp of asthma management, while a score below 18 suggests a need for additional education and support. The AKQ scale has a Cronbach’s alpha coefficient of 0.84 in the original validation study (26), and in the present sample it was 0.81, indicating acceptable reliability.

### 2.8 Quality of life assessment

The Medical Outcomes Study 36-Item Short-Form Health Survey (SF-36), version 2.0, was utilized to assess patients’ quality of life (27). The validated Chinese version (SF-36v2-CN) was administered. The SF-36 comprises 36 items organized into eight domains; for the purposes of this study, we focused on five domains most relevant to asthma outpatients: physical functioning, social functioning, mental health, role limitations due to physical health, and role limitations due to emotional problems. Each domain is scored from 0 to 100 using norm-based scoring, with higher scores indicating better quality of life. The SF-36 demonstrated a Cronbach’s alpha coefficient of 0.814 in the original validation study (27), and in the present sample ranged from 0.78 to 0.88 across domains, indicating good reliability.

### 2.9 Satisfaction

Patient satisfaction was assessed using a hospital-developed Nursing Care Satisfaction Questionnaire (NCSQ). The questionnaire was developed by a multidisciplinary panel comprising three senior nurses, one respiratory physician, and one health services researcher at our institution. Item generation was informed by a literature review and patient focus groups (*n* = 15), and the initial 25-item pool was refined to 20 items following expert panel review for content validity, achieving a content validity index (CVI) of 0.88. The final questionnaire consists of 20 items across four domains: communication quality (5 items), professional competence (5 items), emotional support (5 items), and care environment (5 items). Each item is scored on a 5-point Likert scale (1 = very dissatisfied to 5 = very satisfied), yielding a total score ranging from 20 to 100. A total score below 80 indicates dissatisfaction, 80–95 indicates basic satisfaction, and above 95 indicates full satisfaction. The Cronbach’s alpha for the NCSQ in a pilot sample (*n* = 40) was 0.86, and in the present study sample it was 0.84, indicating good internal consistency. The full questionnaire items are provided in Supplementary Appendix S1.

### 2.10 Statistical method

All continuous variables were tested for normality using the Shapiro–Wilk test. Measurement data conforming to normal distribution are presented as mean ± standard deviation; non-normally distributed data are presented as median (interquartile range). Categorical data are expressed as frequencies and percentages and were compared using the chi-square test or Fisher’s exact test as appropriate. For baseline comparisons between groups, independent-samples *t*-tests were used for normally distributed continuous variables and Mann–Whitney *U* tests for non-normally distributed variables. The primary analysis of post-intervention outcomes employed analysis of covariance (ANCOVA), with post-intervention scores as the dependent variable and the corresponding baseline score as a covariate, to account for any residual baseline differences. To further address potential confounding arising from the non-randomized, self-selected group allocation, multivariable linear regression models were constructed for each continuous outcome, adjusting for age, sex, education level, body mass index, disease duration, asthma severity classification, and baseline score of the respective outcome. For the satisfaction outcome (ordinal categories), ordinal logistic regression was used with the same covariates. Effect sizes were calculated as Cohen’s *d* with 95% confidence intervals for all primary outcomes. A two-sided significance threshold was set at *p* < 0.05. All statistical analyses were performed using SPSS version 26.0 (IBM Corp., Armonk, NY, United States) and R software version 4.2.1 (R Foundation for Statistical Computing, Vienna, Austria).

## 3 Results

### 3.1 Baseline characteristics

The baseline characteristics of participants in the control group (*n* = 71) and the intervention group (*n* = 88) are presented in Table 1. There were no statistically significant differences between groups in age, sex, BMI, education level, marital status, employment status, current smoking, current alcohol consumption, comorbidities (hypertension, diabetes), disease duration, or asthma severity classification according to the Global Initiative for Asthma (GINA) guidelines (20) (all *p* > 0.05). These findings indicate that the two groups were well-matched at baseline.

**Table 1:** Baseline characteristics of participants.

Parameters	Control group (*n* = 71)	Intervention group (*n* = 88)	*t*/*χ*^2^	*p*
Age (years)	43.21 ± 14.87	44.55 ± 14.53	0.571	0.569
Gender (male/female)	36/35	44/44	0.008	0.93
Body mass index (kg/m^2^)	25.64 ± 3.21	25.97 ± 3.18	0.653	0.515
Education level [*n*/(23)]			0.517	0.772
Primary school	12 (16.90%)	18 (20.45%)		
Secondary school	25 (35.21%)	27 (30.68%)		
College	34 (47.89%)	43 (48.86%)		
Marital status [*n*/(23)]			0.472	0.79
Married	50 (70.42%)	66 (75%)		
Single	15 (21.13%)	15 (17.05%)		
Divorced	6 (8.45%)	7 (7.95%)		
Employment [*n* (23)]	49 (69.01%)	61 (69.32%)	0.002	0.967
Current smoking [*n* (%)]	12 (16.9%)	16 (18.18%)	0.044	0.833
Current alcohol consumption [*n* (%)]	8 (11.27%)	12 (13.64%)	0.201	0.654
Hypertension [*n* (23)]	11 (15.49%)	12 (13.64%)	0.109	0.741
Diabetes [*n* (23)]	5 (7.04%)	9 (10.23%)	0.496	0.481
Duration of disease (months)	65.25 ± 21.83	67.59 ± 22.51	0.661	0.51
GINA severity classification			0.126	0.988
Intermittent asthma	20 (28.17%)	27 (30.68%)		
Mild persistent asthma	23 (32.39%)	28 (31.82%)		
Moderate persistent asthma	17 (23.94%)	20 (22.73%)		
Severe persistent asthma	11 (15.49%)	13 (14.77%)		

### 3.2 Lung examinations

Baseline pulmonary function parameters are summarized in Table 2. There were no significant differences between the control and intervention groups in FEV1, FVC, FEV1/FVC ratio, PEFR, or TLC (all *p* > 0.05), confirming comparable lung function at baseline.

**Table 2:** Comparison of lung examinations between two groups of patients.

Parameters	Control group (*n* = 71)	Intervention group (*n* = 88)	*t*	*p*
FEV1 (L)	2.34 ± 0.54	2.38 ± 0.51	0.525	0.6
FVC (L)	3.12 ± 0.78	3.16 ± 0.75	0.302	0.763
FEV1/FVC (23)	75.14 ± 7.23	75.59 ± 7.11	0.397	0.692
PEFR (L/min)	312.25 ± 68.32	316.78 ± 67.41	0.419	0.676
TLC (L)	5.24 ± 1.09	5.29 ± 1.07	0.274	0.784

FEV1, First second forced breathing volume; FVC, forced vital capacity; PEFR, peak expiratory flow rate; TLC, total lung capacity.

### 3.3 Health literacy score before intervention

Baseline health literacy scores are presented in Table 3. The NVS, TOFHLA, HLS-EU, and eHEALS scores were comparable between the control and intervention groups at baseline (all *p* > 0.05), indicating similar health literacy levels prior to the intervention.

**Table 3:** Health literacy score before intervention.

Parameters	Control group (*n* = 71)	Intervention group (*n* = 88)	*t*	*p*
NVS score	4.34 ± 0.62	4.46 ± 0.53	1.297	0.196
TOFHLA score	61.54 ± 6.75	62.98 ± 6.42	1.375	0.171
HLS-EU score	29.83 ± 4.21	30.14 ± 4.13	0.47	0.639
eHEALS score	39.57 ± 5.72	40.86 ± 5.51	1.446	0.15

NVS, Newest Vital Sign; TOFHLA, Test of Functional Health Literacy in Adults; HLS-EU, health literacy survey—European Union; eHEALS, Electronic Health Literacy Scale.

### 3.4 Health literacy score after intervention

**Figure 1:**
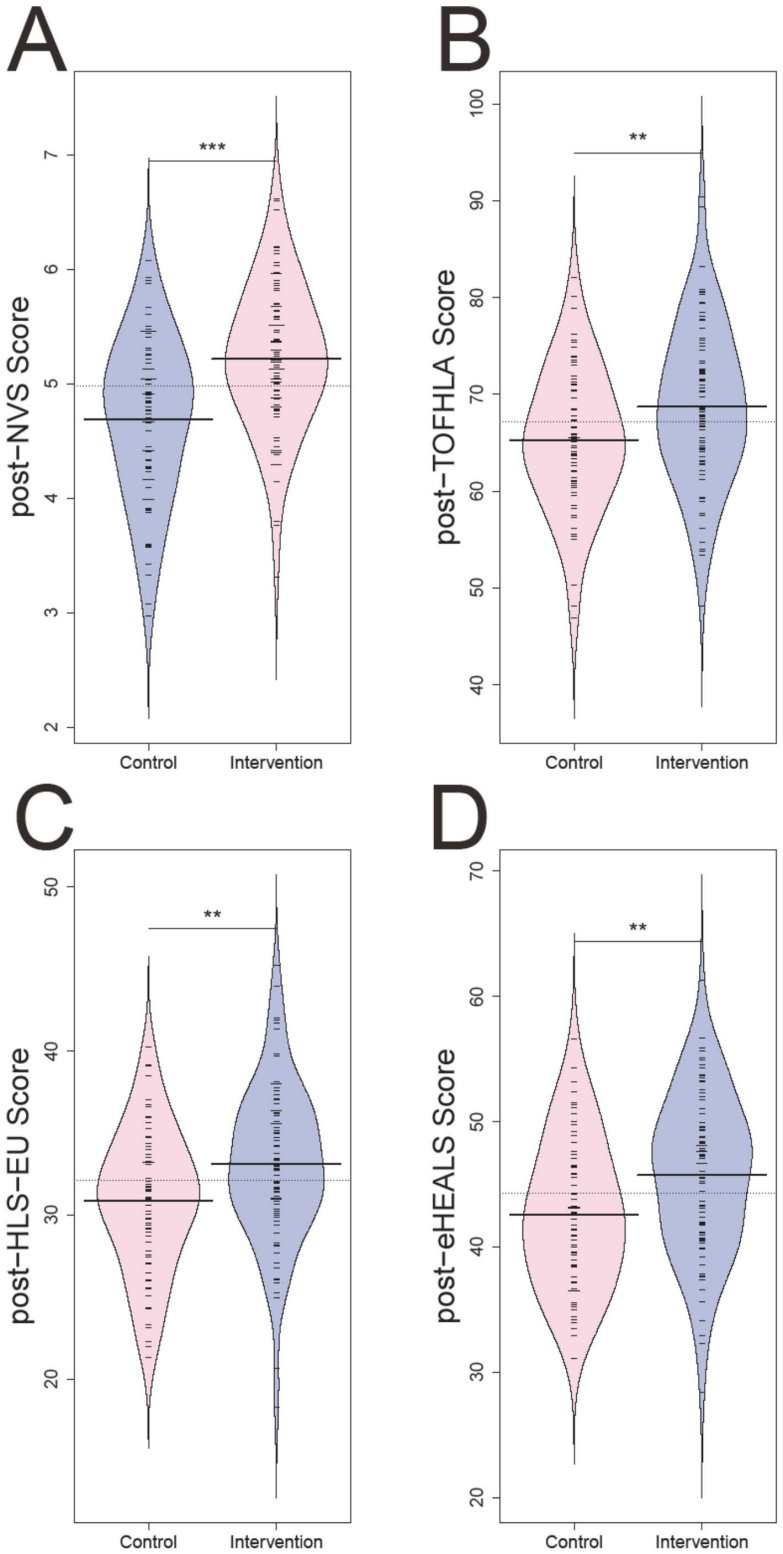
Health literacy score after intervention. **(A)** Post-NVS score; **(B)** Post-TOFHLA score; **(C)** Post-HLS-EU score; **(D)** post-eHEALS score.

Following the intervention, the intervention group demonstrated significantly higher health literacy scores across all measures compared to the control group (Figure 1). After adjusting for baseline values using ANCOVA, the between-group differences remained statistically significant for all health literacy outcomes: NVS (adjusted mean difference 0.51, 95% CI 0.31–0.71, *F* = 24.86, *p* < 0.001, Cohen’s *d* = 0.79, 95% CI 0.47–1.11), TOFHLA (adjusted mean difference 3.28, 95% CI 1.22–5.34, *F* = 9.93, *p* = 0.002, Cohen’s *d* = 0.45, 95% CI 0.14–0.77), HLS-EU (adjusted mean difference 2.18, 95% CI 0.87–3.49, *F* = 10.72, *p* = 0.001, Cohen’s *d* = 0.50, 95% CI 0.18–0.81), and eHEALS (adjusted mean difference 3.04, 95% CI 1.28–4.80, *F* = 11.58, *p* < 0.001, Cohen’s *d* = 0.53, 95% CI 0.21–0.84). After further adjustment for age, sex, education level, BMI, disease duration, and asthma severity in multivariable regression models, the intervention group assignment remained independently associated with higher post-intervention scores for NVS (*β* = 0.48, *p* < 0.001), TOFHLA (*β* = 3.11, *p* = 0.003), HLS-EU (*β* = 2.05, *p* = 0.002), and eHEALS (*β* = 2.87, *p* = 0.001).

### 3.5 Self-care ability scores before intervention

Baseline self-care ability scores are presented in Table 4. There were no significant differences between the control and intervention groups in any of the four ESCA dimensions: self-concept, self-care knowledge, sense of self-preservation, or self-care skills (all *p* > 0.05), indicating comparable baseline levels of self-care abilities.

**Table 4:** Comparison of self-care ability scores between the two groups before intervention.

Parameters	Control group (*n* = 71)	Intervention group (*n* = 88)	*t*	*p*
Self-concept	15.26 ± 4.58	15.82 ± 4.13	0.807	0.421
Self-care knowledge	19.67 ± 6.04	20.18 ± 5.73	0.55	0.583
Sense of self-preservation	15.26 ± 3.91	16.01 ± 3.58	1.272	0.205
Self-care skills	17.28 ± 4.25	18.12 ± 4.34	1.229	0.221

### 3.6 Self-care ability scores after intervention

**Figure 2:**
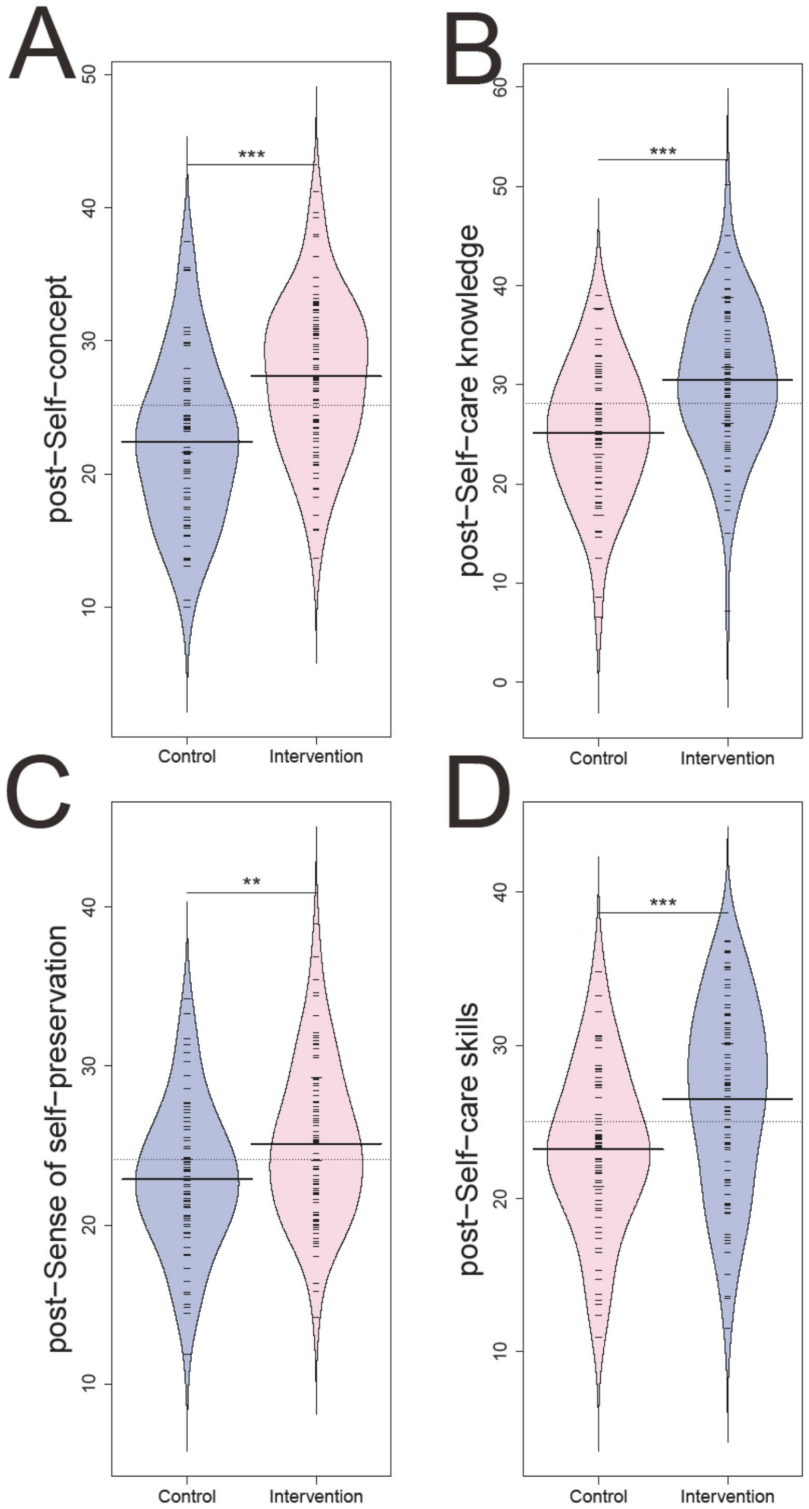
Comparison of self-care ability scores between two groups after intervention. **(A)** Post-Self-concept; **(B)** Post Selfcare-knowledge; **(C)** Post Sense of self preservation; **(D)** Post Selfcare skills.

Post-intervention self-care ability scores are shown in Figure 2. ANCOVA adjusting for baseline values revealed significantly higher scores in the intervention group across all four ESCA dimensions: self-concept (adjusted mean difference 4.72, 95% CI 2.96–6.48, *F* = 28.14, *p* < 0.001, Cohen’s *d* = 0.83, 95% CI 0.51–1.16), self-care knowledge (adjusted mean difference 5.12, 95% CI 3.21–7.03, *F* = 28.02, *p* < 0.001, Cohen’s *d* = 0.76, 95% CI 0.44–1.08), sense of self-preservation (adjusted mean difference 2.14, 95% CI 0.71–3.57, *F* = 8.75, *p* = 0.004, Cohen’s *d* = 0.47, 95% CI 0.16–0.79), and self-care skills (adjusted mean difference 3.15, 95% CI 1.50–4.80, *F* = 14.18, *p* < 0.001, Cohen’s *d* = 0.57, 95% CI 0.25–0.89). After multivariable adjustment, intervention group assignment remained independently associated with higher post-intervention self-concept (*β* = 4.51, *p* < 0.001), self-care knowledge (*β* = 4.88, *p* < 0.001), sense of self-preservation (*β* = 1.98, *p* = 0.006), and self-care skills (*β* = 2.94, *p* < 0.001).

### 3.7 Disease cognition levels

Baseline AKQ scores were comparable between the control group (17.23 ± 3.64) and the intervention group (17.51 ± 3.57; *t* = 0.473, *p* = 0.637) (Table 5). After 12 weeks, ANCOVA adjusting for baseline AKQ scores revealed significantly higher post-intervention AKQ scores in the intervention group compared to the control group (adjusted mean difference 2.25, 95% CI 1.32–3.18, *F* = 22.88, *p* < 0.001, Cohen’s *d* = 0.75, 95% CI 0.43–1.07). Multivariable regression adjusting for age, sex, education level, BMI, disease duration, and asthma severity confirmed the independent association between group assignment and post-intervention AKQ scores (*β* = 2.11, *p* < 0.001).

**Table 5:** Comparison of disease cognition levels between two groups of patients before and after intervention.

Parameters	Control group (*n* = 71)	Intervention group (*n* = 88)	*t*	*p*
Baseline				
AKQ score	17.23 ± 3.64	17.51 ± 3.57	0.473	0.637
12 weeks				
AKQ score	21.12 ± 3.27	23.45 ± 2.92	4.733	<0.001

AKQ, Asthma Knowledge Questionnaire.

### 3.8 Pre-intervention quality of life scores (SF-36)

Baseline SF-36 quality of life scores are presented in Table 6. There were no significant differences between groups in any of the five assessed SF-36 domains: physical functioning, social functioning, mental health, role limitations due to physical health, or role limitations due to emotional problems (all *p* > 0.05).

**Table 6:** Pre-intervention quality of life scores (SF-36).

Parameters	Control group (*n* = 71)	Intervention group (*n* = 88)	*t*	*p*
Physical functioning	65.74 ± 7.83	66.32 ± 7.41	0.48	0.632
Social functioning	68.45 ± 8.26	69.12 ± 8.01	0.519	0.605
Mental health	66.18 ± 6.92	66.75 ± 6.58	0.529	0.598
Role limitations (physical)	63.28 ± 7.54	63.95 ± 7.21	0.569	0.57
Role limitations (emotional)	64.75 ± 6.87	65.32 ± 6.52	0.535	0.593

SF-36, health survey short-form 36.

### 3.9 Post-intervention quality of life scores (SF-36)

Post-intervention SF-36 scores are presented in Table 7. ANCOVA adjusting for baseline values revealed significantly higher scores in the intervention group for all five domains: physical functioning (adjusted mean difference 3.24, 95% CI 1.14–5.34, *F* = 9.26, *p* = 0.003, Cohen’s *d* = 0.47, 95% CI 0.16–0.79), social functioning (adjusted mean difference 3.38, 95% CI 1.22–5.54, *F* = 9.58, *p* = 0.002, Cohen’s *d* = 0.45, 95% CI 0.13–0.76), mental health (adjusted mean difference 3.02, 95% CI 1.15–4.89, *F* = 10.22, *p* = 0.002, Cohen’s *d* = 0.50, 95% CI 0.18–0.81), role limitations due to physical health (adjusted mean difference 3.17, 95% CI 1.08–5.26, *F* = 9.02, *p* = 0.003, Cohen’s *d* = 0.46, 95% CI 0.14–0.77), and role limitations due to emotional problems (adjusted mean difference 2.39, 95% CI 0.48–4.30, *F* = 6.06, *p* = 0.015, Cohen’s *d* = 0.39, 95% CI 0.08–0.71). All associations remained significant after multivariable adjustment for potential confounders.

**Table 7:** Post-intervention quality of life scores (SF-36).

Parameters	Control group (*n* = 71)	Intervention group (*n* = 88)	*t*	*p*
Physical functioning	70.32 ± 7.21	73.68 ± 6.95	2.98	0.003
Social functioning	71.25 ± 7.92	74.75 ± 7.68	2.824	0.005
Mental health	66.63 ± 6.34	69.74 ± 6.18	3.118	0.002
Role limitations (physical)	65.38 ± 7.32	68.65 ± 6.98	2.87	0.005
Role limitations (emotional)	66.45 ± 6.42	68.92 ± 6.18	2.462	0.015

SF-36, health survey short-form 36.

### 3.10 Nursing satisfaction

In the intervention group, 76 patients (86.36%) reported being satisfied or generally satisfied, compared to 46 patients (64.79%) in the control group (*χ*^2^ = 10.244, *p* = 0.001) (Table 8). Ordinal logistic regression adjusting for age, sex, education level, BMI, disease duration, and asthma severity confirmed that intervention group assignment was independently associated with higher satisfaction levels (adjusted OR = 3.42, 95% CI 1.64–7.13, *p* = 0.001).

**Table 8:** Comparison of nursing satisfaction between two groups of patients.

Parameters	Control group (*n* = 71)	Intervention group (*n* = 88)	*χ*^2^	*p*
Satisfied (case)	12	25		
Generally satisfied (case)	34	51		
Dissatisfied (case)	24	12		
Satisfaction [*n* (23)]	46 (64.79%)c	76 (86.36%)	10.244	0.001

## 4 Discussion

In this retrospective cohort study, we observed that nurse-led health guidance was associated with improved self-management skills, disease awareness, and quality of life among outpatients with asthma. The discussion herein aims to elucidate the potential mechanisms underlying these positive associations and highlight the implications of our findings for clinical practice and future research.

The observed association between nurse-led interventions and improved self-management skills may be attributed to several factors. Nursing professionals possess unique expertise in patient education and are well-positioned to bridge the gap between medical care and patient self-management (28). The intervention group received a structured programme of health guidance that included education about disease mechanisms, medication adherence, lifestyle modifications, and coping strategies. Such comprehensive support likely empowered patients to take greater control of their health by enhancing their understanding of asthma and its management. This empowerment is reflected in the significant improvements observed in self-care abilities across all four ESCA dimensions, with medium-to-large effect sizes (Cohen’s *d* ranging from 0.47 to 0.83). Our findings are consistent with a Cochrane systematic review by Gibson et al. (13), which demonstrated that self-management education programmes for adults with asthma improve health outcomes including quality of life, self-efficacy, and reduced healthcare utilization. Similarly, Pinnock et al. (29) found that supported self-management can reduce acute care utilization while improving quality of life. Our results extend this evidence by demonstrating that a nurse-led model of health guidance delivery is associated with improvements across multiple patient-relevant outcome domains simultaneously.

A critical component of the intervention was enhancing patients’ health literacy (30, 31). The nurse-led guidance employed validated tools including the NVS, TOFHLA, HLS-EU, and eHEALS to assess and address patients’ health information competencies. This strategy aligns with existing evidence suggesting that health literacy is a fundamental determinant of self-management and health outcomes (31). By tailoring education to the individual’s literacy levels, nursing staff could effectively communicate complex information in a digestible manner, which may have contributed to improved literacy scores post-intervention. This targeted approach might have also facilitated better medication adherence, a key factor in managing chronic conditions like asthma (32). Importantly, the programme incorporated smoking cessation counselling for patients with a smoking history. Although the present study was not powered to detect differences in smoking cessation rates, this component is supported by strong evidence that smoking cessation is one of the most effective interventions for improving asthma control and long-term respiratory outcomes (8). Future dedicated studies should evaluate the specific impact of nurse-led smoking cessation support on asthma outcomes in this population.

The association between the intervention and increased disease awareness, as evidenced by improved AKQ scores (Cohen’s *d* = 0.75), reflects the centrality of patient education in disease management. The systematic delivery of disease-specific information likely fostered a deeper understanding of asthma, helping patients recognize triggers, symptoms, and appropriate responses. This increased awareness is crucial in enabling patients to anticipate and mitigate exacerbations, thereby maintaining stable health conditions. The positive associations observed echo studies showing that better-informed patients tend to engage more actively in self-care practices, which is in turn associated with improved health outcomes (13, 29).

Furthermore, the improved quality of life observed in the intervention group can be understood through the biopsychosocial model of health. Asthma significantly affects physical functioning, social interaction, and emotional well-being. The multifaceted nature of nurse-led health guidance encompasses psychological support, which may have contributed to the notable improvements in mental health and social functioning scores. Nurses not only provided medical advice but also offered psychological care, addressing anxiety and depression that are often comorbid with chronic conditions (33, 34). This holistic approach likely enhanced patients’ ability to function in daily life, as reflected in their overall quality of life improvements across all five SF-36 domains assessed.

The significant enhancement in nursing satisfaction levels among those receiving nurse-led guidance is reflective of the intervention’s impact on the overall patient experience. Satisfaction is a nuanced outcome reflecting the intersection of clinical effectiveness, care delivery, and personal engagement. The ordinal logistic regression analysis confirmed that this association was independent of demographic and clinical confounders (adjusted OR = 3.42, 95% CI 1.64–7.13). Higher satisfaction is often linked with improved compliance, reduced anxiety, and increased trust in healthcare providers, forming a positive feedback loop that supports ongoing health management and positive outcomes.

Despite the study’s positive findings, it is essential to consider the mechanisms of change within the broader healthcare system context. The nurse-led model of care might promote a more collaborative interdisciplinary approach wherein nurses serve as primary educators and care facilitators, potentially relieving physicians’ workloads and encouraging more personalized patient interactions. This system-level integration could streamline patient education, leading to consistent messaging and improved care continuity.

Additionally, the intervention group’s improved outcomes may be partly attributed to the enhanced patient-provider relationship fostered by regular, structured interactions. Frequent contact with healthcare providers through scheduled nurse-led sessions may have built rapport that encouraged patients to express concerns and seek clarification, further enhancing their confidence in self-management and disease understanding. This relational component is frequently underestimated yet essential in chronic disease management (35).

This study has several limitations that warrant consideration. First, the retrospective, non-randomized design introduces the possibility of selection bias and residual confounding, as participants self-selected their health guidance model. Although we employed ANCOVA and multivariable regression to adjust for measured confounders, unmeasured confounders such as health motivation, socioeconomic status, and baseline treatment adherence may have influenced the observed associations. Future prospective randomized controlled trials are needed to establish causality. Second, the sample size of 159 participants from a single centre limits the generalizability of the findings to other settings and populations. Third, the 12-week follow-up period was relatively short; longer-term follow-up would be valuable to determine whether the observed benefits are sustained and to assess the impact on asthma-specific clinical outcomes such as exacerbation rates, Asthma Control Test (ACT) scores, and healthcare utilization, which were not captured in the present study. Fourth, we did not assess asthma-specific outcomes including exacerbation frequency, inhaler technique proficiency, or adherence rates, which would have provided a more comprehensive evaluation of the intervention’s clinical impact. Fifth, the satisfaction questionnaire was hospital-developed and, while demonstrating acceptable content validity and internal consistency, has not undergone extensive external validation. Finally, assessment of outcomes relied on self-report questionnaires, which are subject to response and social desirability bias.

Given these insights, the implications for clinical practice merit consideration. Implementing nurse-led health guidance as a complementary approach to conventional treatments may be a viable strategy to enhance patient engagement, improve adherence, and ultimately contribute to better long-term health outcomes. The adoption of such models requires shifts in organizational culture and practice patterns, underscoring the need for healthcare systems to recognize the multifaceted roles of nursing professionals.

Future research should aim to explore the cost-effectiveness of nurse-led interventions through prospective randomized controlled trials with adequate sample sizes and longer follow-up periods. Studies should incorporate asthma-specific clinical endpoints including exacerbation rates, ACT scores, peak flow variability, and healthcare utilization. Additionally, investigating the specific components of nurse-led guidance that yield the most significant improvements through dismantling study designs would be valuable in refining and optimizing these interventions. The applicability of the model in other chronic disease contexts, as well as the integration of digital health technologies to augment nurse-led care, also deserves further investigation.

## 5 Conclusion

In conclusion, this retrospective cohort study demonstrates that nurse-led health guidance is associated with significantly improved self-management skills, disease awareness, and quality of life in asthma outpatients. These associations persisted after statistical adjustment for potential confounders. The findings support the integration of nurse-led models into chronic disease management strategies and warrant confirmation through prospective randomized controlled trials. By leveraging the unique skills and relational capabilities of nurses, healthcare systems may potentially improve patient experiences and outcomes in meaningful ways.

## Data Availability

The original contributions presented in the study are included in the article/supplementary material, further inquiries can be directed to the corresponding author.
