# Supplementary material for: The effectiveness of nurse led health guidance in improving self-management skills, disease awareness, and quality of life for outpatient patients
Source: Front Med (Lausanne). 2026 Jul 16;13:1774746. doi: 10.3389/fmed.2026.1774746 (PMC13422209; doi:10.3389/fmed.2026.1774746)
Supplement: Supplementary file 1 [file Supplementary_file_1.docx]

**Supplementary Appendix S1**

**Nursing Care Satisfaction Questionnaire (NCSQ)**

*for outpatient patients with asthma*

**Instructions:** Please rate your satisfaction with the nursing care and health guidance received during outpatient asthma management. Select one response for each item according to your actual experience.

**Response options:** 1 = very dissatisfied; 2 = dissatisfied; 3 = neutral; 4 = satisfied; 5 = very satisfied.

**Scoring:** The questionnaire includes 20 items across four domains. Each item is scored from 1 to 5, yielding a total score from 20 to 100. A total score below 80 indicates dissatisfaction, 80–95 indicates basic satisfaction, and above 95 indicates full satisfaction.

| **Domain** | **No.** | **Item** | **1** | **2** | **3** | **4** | **5** |
| --- | --- | --- | --- | --- | --- | --- | --- |
| Communication quality | 1 | The nurse explained my condition and care plan in language that I could understand. |  |  |  |  |  |
| Communication quality | 2 | The nurse listened carefully to my questions and concerns. |  |  |  |  |  |
| Communication quality | 3 | The nurse provided clear guidance about asthma self-management. |  |  |  |  |  |
| Communication quality | 4 | The nurse gave timely answers during clinic visits or follow-up calls. |  |  |  |  |  |
| Communication quality | 5 | The nurse communicated with me respectfully and patiently. |  |  |  |  |  |
| Professional competence | 6 | The nurse demonstrated professional knowledge about asthma care. |  |  |  |  |  |
| Professional competence | 7 | The nurse correctly explained medication use and possible adverse effects. |  |  |  |  |  |
| Professional competence | 8 | The nurse demonstrated inhaler technique clearly and checked my return demonstration. |  |  |  |  |  |
| Professional competence | 9 | The nurse helped me recognize warning signs of asthma exacerbation. |  |  |  |  |  |
| Professional competence | 10 | The nurse provided practical advice tailored to my health status. |  |  |  |  |  |
| Emotional support | 11 | The nurse showed concern for my feelings and difficulties. |  |  |  |  |  |
| Emotional support | 12 | The nurse helped reduce my anxiety about asthma management. |  |  |  |  |  |
| Emotional support | 13 | The nurse encouraged me to build confidence in self-care. |  |  |  |  |  |
| Emotional support | 14 | The nurse respected my preferences when discussing care choices. |  |  |  |  |  |
| Emotional support | 15 | The nurse provided support when I had difficulty following the care plan. |  |  |  |  |  |
| Care environment | 16 | The nursing service process was organized and convenient. |  |  |  |  |  |
| Care environment | 17 | The counselling environment protected my privacy. |  |  |  |  |  |
| Care environment | 18 | The outpatient nursing area was clean and comfortable. |  |  |  |  |  |
| Care environment | 19 | The follow-up arrangements were convenient and reliable. |  |  |  |  |  |
| Care environment | 20 | Overall, the nursing care environment met my needs. |  |  |  |  |  |

**Note:** This appendix corresponds to the patient satisfaction assessment described in Section 2.9 of the manuscript.
